# The Transcription Factor Ndt80 Does Not Contribute to Mrr1-, Tac1-, and Upc2-Mediated Fluconazole Resistance in *Candida albicans*

**DOI:** 10.1371/journal.pone.0025623

**Published:** 2011-09-27

**Authors:** Christoph Sasse, Rebecca Schillig, Franziska Dierolf, Michael Weyler, Sabrina Schneider, Selene Mogavero, P. David Rogers, Joachim Morschhäuser

**Affiliations:** 1 Institut für Molekulare Infektionsbiologie, Universität Würzburg, Würzburg, Germany; 2 Department of Biology, University of Pisa, Pisa, Italy; 3 Children's Foundation Research Center, Le Bonheur Children's Hospital, Memphis, Tennessee, United States of America; 4 Department of Pharmaceutical Sciences, College of Pharmacy, University of Tennessee Health Science Center, Memphis, Tennessee, United States of America; 5 Department of Clinical Pharmacy, College of Pharmacy, University of Tennessee Health Science Center, Memphis, Tennessee, United States of America; 6 Department of Pediatrics, College of Medicine, University of Tennessee Health Science Center, Memphis, Tennessee, United States of America; Newcastle University, United Kingdom

## Abstract

The pathogenic yeast *Candida albicans* can develop resistance to the widely used antifungal agent fluconazole, which inhibits ergosterol biosynthesis, by the overexpression of genes encoding multidrug efflux pumps or ergosterol biosynthesis enzymes. Zinc cluster transcription factors play a central role in the transcriptional regulation of drug resistance. Mrr1 regulates the expression of the major facilitator *MDR1*, Tac1 controls the expression of the ABC transporters *CDR1* and *CDR2*, and Upc2 regulates ergosterol biosynthesis (*ERG*) genes. Gain-of-function mutations in these transcription factors result in constitutive overexpression of their target genes and are responsible for fluconazole resistance in many clinical *C. albicans* isolates. The transcription factor Ndt80 contributes to the drug-induced upregulation of *CDR1* and *ERG* genes and also binds to the *MDR1* and *CDR2* promoters, suggesting that it is an important component of all major transcriptional mechanisms of fluconazole resistance. However, we found that Ndt80 is not required for the induction of *MDR1* and *CDR2* expression by inducing chemicals. *CDR2* was even partially derepressed in *ndt80*Δ mutants, indicating that Ndt80 is a repressor of *CDR2* expression. Hyperactive forms of Mrr1, Tac1, and Upc2 promoted overexpression of *MDR1*, *CDR1*/*CDR2*, and *ERG11*, respectively, with the same efficiency in the presence and absence of Ndt80. Mrr1- and Tac1-mediated fluconazole resistance was even slightly enhanced in *ndt80*Δ mutants compared to wild-type cells. These results demonstrate that Ndt80 is dispensable for the constitutive overexpression of Mrr1, Tac1, and Upc2 target genes and the increased fluconazole resistance of strains that have acquired activating mutations in these transcription factors.

## Introduction

Infections by the pathogenic yeast *Candida albicans* are commonly treated with the antifungal agent fluconazole, which blocks ergosterol biosynthesis by inhibiting sterol 14α-demethylase, a key enzyme in the ergosterol biosynthetic pathway. *C. albicans* can develop resistance to fluconazole by various mechanisms, including mutations in the target enzyme that decrease its affinity to the drug, increased expression of the *ERG11* gene encoding sterol 14α-demethylase, or overexpression of multidrug efflux pumps of the ABC transporter and major facilitator superfamilies [1]. Many details of the molecular basis of drug resistance in *C. albicans* have been elucidated in recent years, especially with the identification of transcription factors that regulate the expression of ergosterol biosynthesis genes and multidrug efflux pumps. Transcription factors of the zinc cluster family, which is specific for fungi, have central roles in the transcriptional control of fluconazole resistance in *C. albicans*. Upc2 regulates the expression of *ERG11* and other *ERG* genes [2], [3], Tac1 controls the expression of the ABC transporters *CDR1* and *CDR2* [4], and Mrr1 regulates the expression of the major facilitator *MDR1* [5]. Mutants lacking these transcription factors cannot upregulate their target genes in response to inducing stimuli. In addition, fluconazole-resistant, clinical *C. albicans* isolates that overexpress *CDR1* and *CDR2*, *MDR1*, or *ERG11* contain gain-of-function mutations in Tac1, Mrr1, and Upc2, respectively, which render the transcription factors constitutively active even under noninducing conditions and are responsible for the increased drug resistance of these strains [4], [5], [6], [7], [8], [9], [10], [11], [12], [13], [14].

While Mrr1, Tac1, and Upc2 each contribute in different and specific ways to the development of fluconazole resistance, another transcription factor, Ndt80, seems to be involved in the transcriptional regulation of Mrr1, Tac1, and Upc2 target genes and therefore have a much broader role in fluconazole resistance and many other cellular functions in *C. albicans* [15], [16]. *NDT80* was originally identified as a gene that increased *CDR1* promoter activity when it was overexpressed from a multicopy plasmid in the heterologous host *Saccharomyces cerevisiae* [17]. Deletion of *NDT80* in *C. albicans* resulted in reduced basal *CDR1* expression levels, and induction of *CDR1* expression by miconazole was abolished in the *ndt80*Δ mutants, which became hypersusceptible to fluconazole and voriconazole [17], [18]. Expression of *NDT80* itself was found to be induced upon treatment of *C. albicans* cells with itraconazole or miconazole [17], [19]. The involvement of Ndt80 in *CDR1* regulation was corroborated by the finding that Ndt80 binds to the *CDR1* promoter in vivo [16]. Interestingly, Ndt80 also binds to the promoters of the efflux pumps *CDR2* and *MDR1* and those of genes encoding ergosterol biosynthesis enzymes, including *ERG11* [16]. Ndt80 therefore seems to be involved in the transcriptional regulation of all major mechanisms of azole resistance in *C. albicans*.

While a role of Ndt80 in *CDR1* expression and fluconazole-induced upregulation of *ERG* genes has been demonstrated [16], [17], the relevance of Ndt80 binding to the promoters of *MDR1* and *CDR2* for the expression of these efflux pumps has not yet been addressed. We therefore investigated if Ndt80 is required for *MDR1* and *CDR2* upregulation by chemicals that are known to induce the expression of these drug transporters. In addition, we studied the importance of Ndt80 for the constitutive overexpression of *MDR1*, *CDR1*/*CDR2*, and *ERG11* in strains containing hyperactive *MRR1*, *TAC1*, and *UPC2* alleles, respectively, which confer increased fluconazole resistance in clinical *C. albicans* isolates.

## Results

### Role of Ndt80 in *MDR1* induction and constitutive *MDR1* overexpression

**Figure 1:**
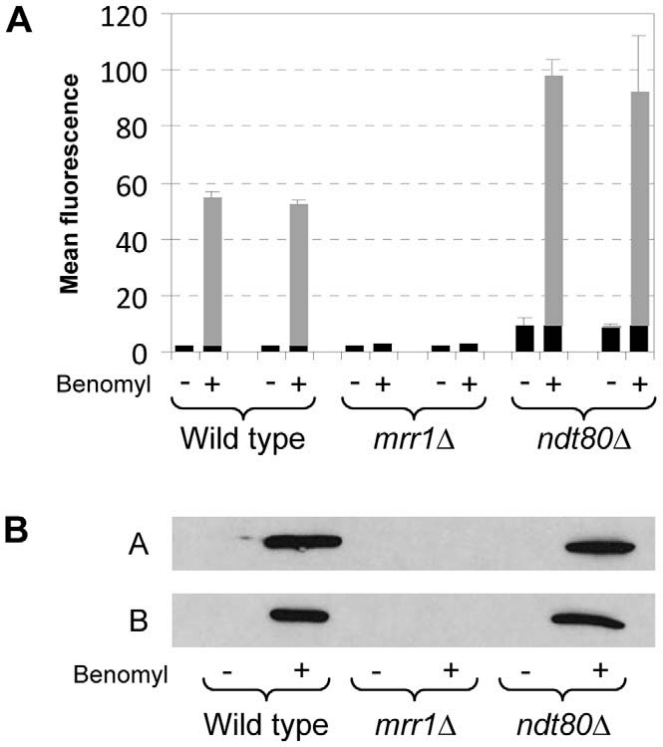
Inducibility of the *MDR1* promoter in wild-type, *mrr1*Δ, and *ndt80*Δ strains. (A) Strains carrying a P*_MDR1_-GFP* reporter fusion in the indicated genetic backgrounds were grown in the absence (−) or presence (+) of benomyl as described in Materials and methods. The mean fluorescence of the cells was determined by flow cytometry. The results obtained with two independently generated reporter strains are shown in each case (means and standard deviations from three experiments). The following strains were used (see Table S1): SCMG3A and -B (wild type), SCΔ*mrr1*MG3A and -B (*mrr1*Δ), SCΔ*ndt80*MG3A and -B (*ndt80*Δ). The background fluorescence of the parental strains, which do not contain the *GFP* gene, is indicated by the black part in each column. (B) GFP expression in the same strains was detected by Western immunoblotting with an anti-GFP antibody. The two blots show the results with the A and B series of strains.

To evaluate the importance of Ndt80 for *MDR1* expression, we constructed two independent *ndt80*Δ mutants of the wild-type strain SC5314 by the *SAT1* flipping method (see Materials and methods). We then integrated the *GFP* reporter gene under the control of the native *MDR1* promoter in the wild type and the *ndt80*Δ mutants to quantify *MDR1* promoter activity. For comparison, the reporter fusion was also introduced in two independently generated *mrr1*Δ mutants of strain SC5314. The *MDR1* gene is not significantly expressed under standard growth conditions, but it is activated in the presence of benomyl [5], [20], [21]. FACS analysis of the cells showed that Ndt80 was not required for the induction of the *MDR1* promoter by benomyl, whereas *MDR1* induction was abolished in cells lacking Mrr1 (Figure 1A). As the *ndt80*Δ mutants displayed a higher background fluorescence than the wild type, we also detected GFP expression by Western immunoblotting (Figure 1B). These experiments confirmed that comparable amounts of GFP were produced in the presence and absence of *NDT80*, i.e., Ndt80 was dispensable for the induction of the *MDR1* promoter by benomyl.

**Figure 2:**
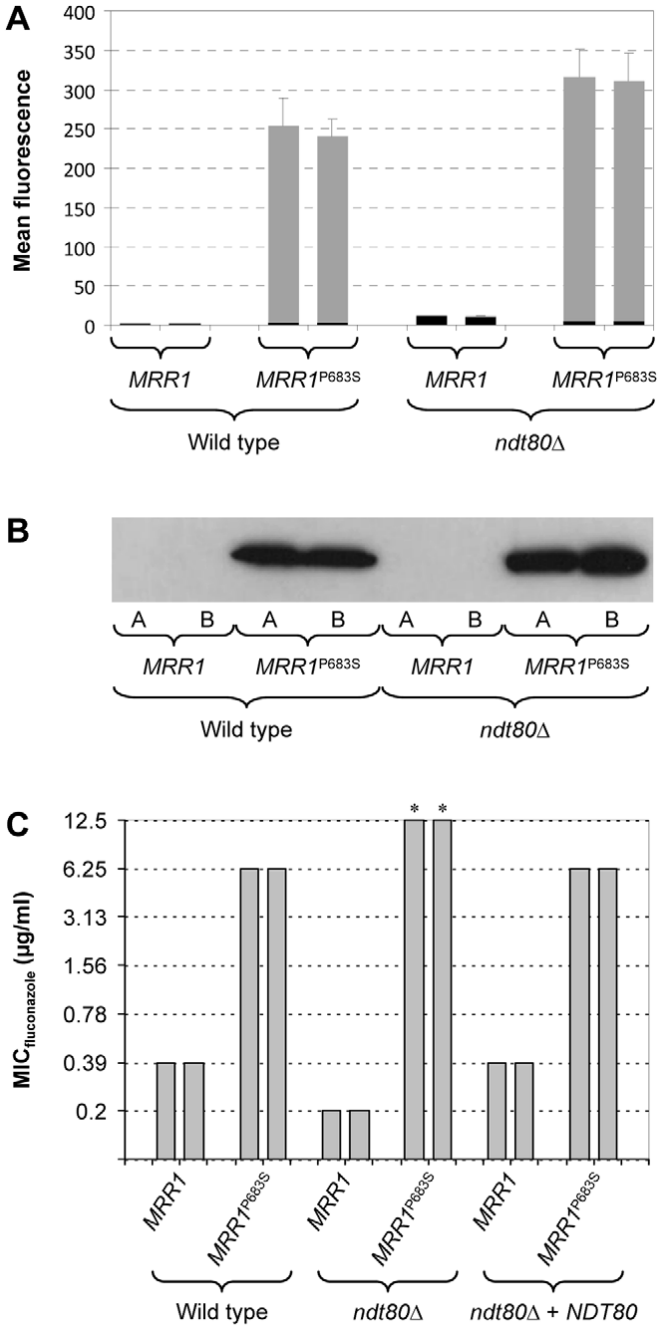
*MDR1* promoter activity and fluconazole resistance of strains expressing the hyperactive *MRR1* ^P683S^ allele in wild-type and *ndt80*Δ backgrounds. (A) Reporter strains containing wild-type or hyperactive *MRR1* alleles and expressing *GFP* under the control of the *MDR1* promoter were grown to log phase in YPD medium. The mean fluorescence of the cells was determined by flow cytometry. The results obtained with two independently generated reporter strains are shown in each case (means and standard deviations from three experiments). The following strains were used (see Table S1): SCMG3A and -B (wild type, *MRR1*), SCMRR1R34MG3A and -B (wild type, *MRR1* ^P683S^), SCΔ*ndt80*MG3A and -B (*ndt80*Δ, *MRR1*), SCΔ*ndt80*MRR1R34MG3A and -B (*ndt80*Δ, *MRR1* ^P683S^). The background fluorescence of the parental strains, which do not contain the *GFP* gene, is indicated by the black part in each column. (B) GFP expression in the same strains was detected by Western immunoblotting with an anti-GFP antibody. (C) MIC of fluconazole for strains containing wild-type *MRR1* or *MRR1* ^P683S^ alleles in the indicated genetic backgrounds. *, reduced growth was already observed at 6.25 µg/ml fluconazole.

A more important question with respect to drug resistance was whether Ndt80 contributes to the constitutive *MDR1* overexpression in strains containing a hyperactive Mrr1. To address this issue, we introduced a gain-of-function mutation that causes a P683S amino acid substitution into both endogenous *MRR1* alleles of the wild-type strain SC5314 and the *ndt80*Δ mutants. The P*_MDR1_-GFP* reporter fusion was then integrated into these strains to compare their *MDR1* promoter activities. FACS and Western immunoblot analysis of GFP expression demonstrated that the hyperactive Mrr1 induced the *MDR1* promoter with similar efficiency in both strain backgrounds (Figure 2A and B).

To evaluate the phenotypic consequences of expression of a hyperactive Mrr1 in the absence of Ndt80, we determined the fluconazole susceptibilities of wild-type and *ndt80*Δ strains containing native or hyperactive *MRR1* alleles (Figure 2C). In line with previous results [22], expression of the hyperactive *MRR1* ^P683S^ allele in the wild-type background resulted in a 16-fold increase in fluconazole resistance (MIC increased from 0.39 to 6.25 µg/ml). In accord with findings by other researchers [16], [17], we observed an enhanced fluconazole sensitivity of the *ndt80*Δ mutants. Surprisingly, however, the presence of the hyperactive *MRR1* ^P683S^ allele caused an even stronger increase in fluconazole resistance in the *ndt80*Δ mutants (MIC increased from 0.2 to 12.5 µg/ml) than in their wild-type parent. To confirm that this phenotype was indeed caused by the deletion of *NDT80*, we reinserted a functional *NDT80* copy into the homozygous *ndt80*Δ mutants. The reintroduction of *NDT80* reverted the fluconazole susceptibilities of the *ndt80*Δ mutants expressing native of hyperactive *MRR1* alleles to the levels observed in the corresponding wild-type strains.

Altogether, these results demonstrated that Ndt80 is dispensable for *MDR1* upregulation in response to the inducer benomyl and for the constitutive *MDR1* overexpression caused by a hyperactive Mrr1. In addition, Ndt80 has a negative effect on Mrr1-mediated fluconazole resistance, presumably by reducing the expression of other Mrr1 target genes that also contribute to drug resistance [22].

### Role of Ndt80 in *CDR2* induction and constitutive *CDR2* overexpression

**Figure 3:**
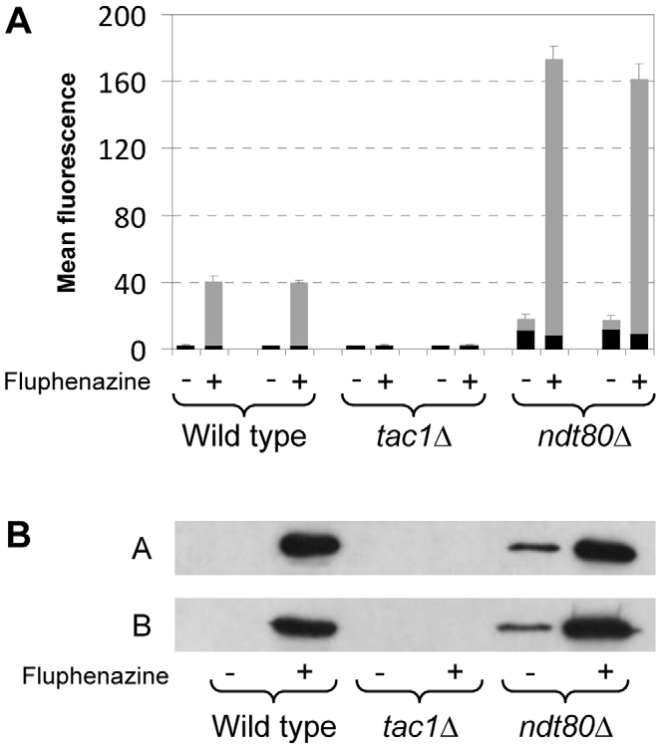
Inducibility of the *CDR2* promoter in wild-type, *tac1*Δ, and *ndt80*Δ strains. (A) Strains carrying a P*_CDR2_-GFP* reporter fusion in the indicated genetic backgrounds were grown in the absence (−) or presence (+) of fluphenazine as described in Materials and methods. The mean fluorescence of the cells was determined by flow cytometry. The results obtained with two independently generated reporter strains are shown in each case (means and standard deviations from three experiments). The following strains were used (see Table S1): SCCG3A and -B (wild type), SCΔ*tac1*CG3A and -B (*tac1*Δ), SCΔ*ndt80*CG3A and -B (*ndt80*Δ). The background fluorescence of the parental strains, which do not contain the *GFP* gene, is indicated by the black part in each column. (B) GFP expression in the same strains was detected by Western immunoblotting with an anti-GFP antibody. The two blots show the results with the A and B series of strains.

As explained in the introduction, Ndt80 also binds to the promoter of the ABC transporter *CDR2*, which like *CDR1* is regulated by the zinc cluster transcription factor Tac1. *CDR2* is not significantly expressed under standard growth conditions, but its expression is strongly induced by fluphenazine [21], [23], [24]. To investigate if Ndt80 is required for the activation of the *CDR2* promoter in response to this compound, we integrated a P*_CDR2_-GFP* reporter fusion into the endogenous *CDR2* locus of the wild-type strain SC5314 and the *ndt80*Δ mutants. For comparison, the reporter fusion was also introduced in two independently generated *tac1*Δ mutants of strain SC5314. FACS analysis of the cells (Figure 3A) and Western immunoblot analysis of GFP expression (Figure 3B) demonstrated that the *CDR2* promoter was efficiently induced by fluphenazine in both the wild type and the *ndt80*Δ mutants, while no induction was observed in *tac1*Δ mutants, in agreement with previous findings [4]. Interestingly, the *ndt80*Δ mutants also exhibited a basal *CDR2* promoter activity in the absence of the inducer, in contrast to the wild type, in which no *CDR2* expression was detectable under noninducing conditions. These results indicated that Ndt80 is a repressor of *CDR2* expression.

**Figure 4:**
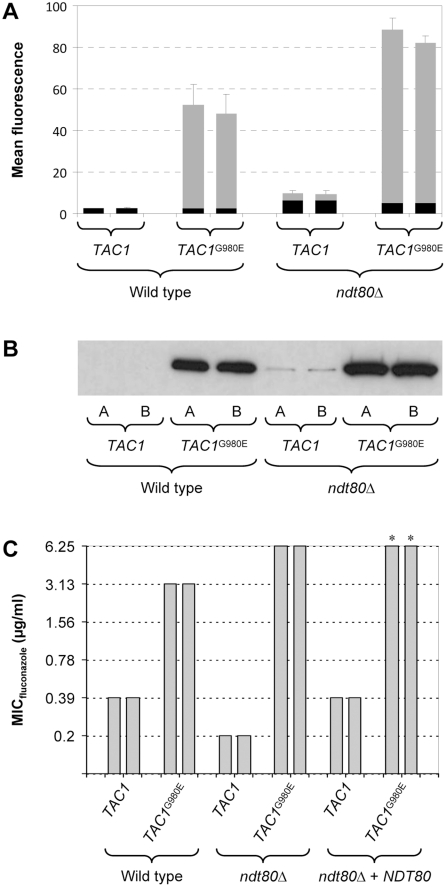
*CDR2* promoter activity and fluconazole resistance of strains expressing the hyperactive *TAC1* ^G980E^ allele in wild-type and *ndt80*Δ backgrounds. (A) Reporter strains containing wild-type or hyperactive *TAC1* alleles and expressing *GFP* under the control of the *CDR2* promoter were grown to log phase in YPD medium. The mean fluorescence of the cells was determined by flow cytometry. The results obtained with two independently generated reporter strains are shown in each case (means and standard deviations from three experiments). The following strains were used (see Table S1): SCCG3A and -B (wild type, *TAC1*), SCTAC1R34CG3A and -B (wild type, *TAC1* ^G980E^), SCΔ*ndt80*CG3A and -B (*ndt80*Δ, *TAC1*), SCΔ*ndt80*TAC1R34CG3A and -B (*ndt80*Δ, *TAC1* ^G980E^). The background fluorescence of the parental strains, which do not contain the *GFP* gene, is indicated by the black part in each column. (B) GFP expression in the same strains was detected by Western immunoblotting with an anti-GFP antibody. (C) MIC of fluconazole for strains containing wild-type *TAC1* or *TAC1* ^G980E^ alleles in the indicated genetic backgrounds. *, reduced growth was already observed at 3.13 µg/ml fluconazole.

Activating mutations in Tac1 result in constitutive *CDR2* overexpression in fluconazole-resistant strains. To investigate whether Ndt80 affects *CDR2* induction by a hyperactive Tac1, we introduced a gain-of-function mutation that results in a G980E amino acid substitution into both endogenous *TAC1* alleles of the wild-type strain SC5314 and the *ndt80*Δ mutants. The P*_CDR2_-GFP* reporter fusion was then integrated into these strains to compare their *CDR2* promoter activities. FACS analysis of the cells (Figure 4A) and Western immunoblot analysis of GFP expression (Figure 4B) demonstrated that the hyperactive Tac1 induced the *CDR2* promoter in the *ndt80*Δ mutants with similar efficiency as in wild-type cells.

In order to determine if a hyperactive Tac1 can mediate increased drug resistance in *ndt80*Δ mutants, we compared the fluconazole susceptibilities of wild-type and *ndt80*Δ strains containing native or hyperactive *TAC1* alleles (Figure 4C). In the wild-type background, the *TAC1* ^G980E^ allele caused an 8-fold increased fluconazole resistance (MIC increased from 0.39 to 3.13 µg/ml). An even stronger increase in resistance (32-fold) was observed in the *ndt80*Δ mutants, in which the MIC rose from 0.2 to 6.25 µg/ml. When a single *NDT80* copy was reinserted into the homozygous *ndt80*Δ mutants containing the *TAC1* ^G980E^ alleles, the MIC of fluconazole remained at 6.25 µg/ml, but the strains showed reduced growth at 3.13 µg/ml, indicating that their resistance was intermediate between that of cells containing two *NDT80* copies or none. These results demonstrated that Ndt80 also has a negative effect on Tac1-mediated fluconazole resistance.

### Ndt80 is not required for Tac1-mediated *CDR1* overexpression

**Figure 5:**
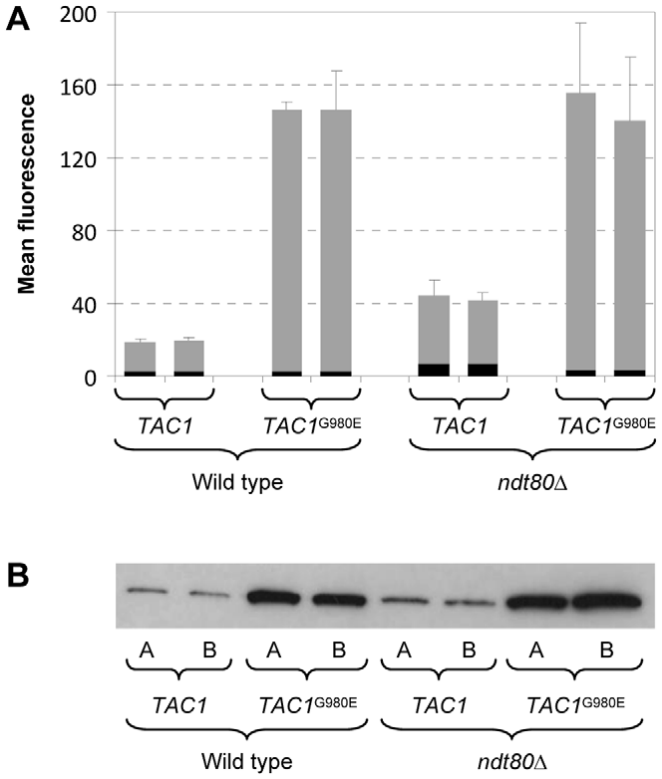
*CDR1* promoter activity in strains expressing the hyperactive *TAC1* ^G980E^ allele in wild-type and *ndt80*Δ backgrounds. (A) Reporter strains containing wild-type or hyperactive *TAC1* alleles and expressing *GFP* under the control of the *CDR1* promoter were grown to log phase in YPD medium. The mean fluorescence of the cells was determined by flow cytometry. The results obtained with two independently generated reporter strains are shown in each case (means and standard deviations from three experiments). The following strains were used (see Table S1): SCCG1A and -B (wild type, *TAC1*), SCTAC1R34CG1A and -B (wild type, *TAC1* ^G980E^), SCΔ*ndt80*CG1A and -B (*ndt80*Δ, *TAC1*), SCΔ*ndt80*TAC1R34CG1A and -B (*ndt80*Δ, *TAC1* ^G980E^). The background fluorescence of the parental strains, which do not contain the *GFP* gene, is indicated by the black part in each column. (B) GFP expression in the same strains was detected by Western immunoblotting with an anti-GFP antibody.

*CDR1* and *CDR2* are usually coregulated by Tac1 [4], [6], [7], [14]. In the light of previous reports that Ndt80 is an activator of *CDR1* expression [17], [18], our findings that *NDT80* is not required for the inducible or constitutive upregulation of *CDR2* and even inhibits *CDR2* expression under noninducing conditions therefore came as a surprise. As *CDR1* makes a stronger contribution than *CDR2* to the increased drug resistance of strains in which these ABC transporters are overexpressed [25], [26], we compared the *CDR1* promoter activities of strains containing native or hyperactive *TAC1* alleles in wild-type and *ndt80*Δ backgrounds. As can be seen in Figure 5, the hyperactive Tac1 caused a similar constitutive *CDR1* overexpression in the presence and absence of Ndt80, explaining our finding that Ndt80 is not required for Tac1-mediated fluconazole resistance.

### Ndt80 is dispensable for ERG11 upregulation by a hyperactive Upc2

**Figure 6:**
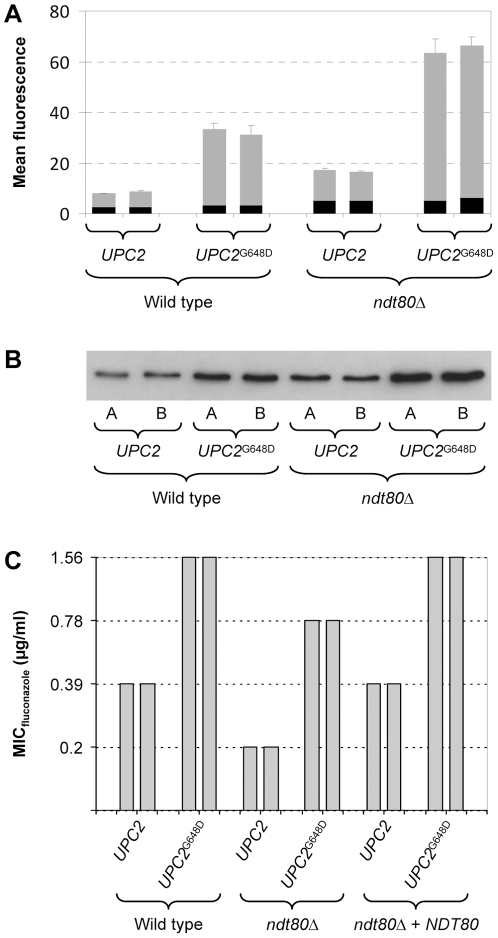
*ERG11* promoter activity and fluconazole resistance of strains expressing the hyperactive *UPC2* ^G648D^ allele in wild-type and *ndt80*Δ backgrounds. (A) Reporter strains containing wild-type or hyperactive *UPC2* alleles and expressing *GFP* under the control of the *ERG11* promoter were grown to log phase in YPD medium. The mean fluorescence of the cells was determined by flow cytometry. The results obtained with two independently generated reporter strains are shown in each case (means and standard deviations from three experiments). The following strains were used (see Table S1): SCEG2A and -B (wild type, *UPC2*), SCUPC2R14CG2A and -B (wild type, *UPC2* ^G648D^), SCΔ*ndt80*EG2A and -B (*ndt80*Δ, *UPC2*) SCΔ*ndt80*UPC2R14EG2A and -B (*ndt80*Δ, *UPC2* ^G648D^). The background fluorescence of the parental strains, which do not contain the *GFP* gene, is indicated by the black part in each column. (B) GFP expression in the same strains was detected by Western immunoblotting with an anti-GFP antibody. (C) MIC of fluconazole for strains containing wild-type *UPC2* or *UPC2* ^G648D^ alleles in the indicated genetic backgrounds.

Ndt80 has been found to contribute to the fluconazole-induced upregulation of ergosterol biosynthesis genes, including *ERG11* [16]. Gain-of-function mutations in the zinc cluster transcription factor Upc2 result in constitutive *ERG11* overexpression and increased fluconazole resistance [10], [11], [12]. To evaluate the importance of Ndt80 for the Upc2-mediated constitutive *ERG11* upregulation, we introduced a gain-of-function mutation that results in a G648D amino acid substitution into both endogenous *UPC2* alleles of the wild-type strain SC5314 and the *ndt80*Δ mutants. The P*_ERG11_-GFP* reporter fusion was then integrated into these strains to compare their *ERG11* promoter activities. FACS analysis of the cells (Figure 6A) and Western immunoblot analysis of GFP expression (Figure 6B) demonstrated that the hyperactive Upc2 caused a constitutive *ERG11* overexpression in the presence and absence of Ndt80. To assess whether the hyperactive Upc2 could mediate increased fluconazole resistance in *ndt80*Δ mutants, we determined the MIC of fluconazole for the strains. Expression of the hyperactive Upc2 resulted in a 4-fold increase in fluconazole resistance in both the wild type and the *ndt80*Δ mutants, although the hypersusceptibility of the *ndt80*Δ mutants as compared with the wild type was conserved in the presence of the *UPC2* ^G648D^ allele (Figure 6C). Reintroduction of a single copy of *NDT80* in the *ndt80*Δ mutants restored fluconazole resistance to the level of the corresponding wild-type strains. These results demonstrate that Ndt80 is not required for the constitutive *ERG11* overexpression and the resulting increase in fluconazole resistance caused by a hyperactive Upc2.

## Discussion

Previous findings that Ndt80 contributes to the drug-induced upregulation of *CDR1* and *ERG11* and that it also binds to the promoters of *MDR1* and *CDR2* suggested that Ndt80 is involved in all major transcriptional mechanisms of fluconazole resistance in *C. albicans* [16], [17]. We found that Ndt80 was dispensable for the upregulation of *MDR1* in response to the inducer benomyl and for the induction of *CDR2* expression by fluphenazine. In fact, *CDR2* expression was partially derepressed in *ndt80*Δ mutants, arguing that Ndt80 binds to the *CDR2* promoter to act as a repressor of this efflux pump under noninducing conditions. In contrast to a previous report [17], we did not observe a reduced basal expression of *CDR1* in the absence of Ndt80 (Figure 5). However, this result concurs with another study in which *CDR1* was not found among the genes that were differentially expressed in an *ndt80*Δ mutant [16].

It is likely that Ndt80 is required only under specific conditions for the expression of the genes to which it binds. Ndt80 was shown to bind to the promoter regions of 23% of *C. albicans* genes, but the expression of many of these genes was not altered after deletion of *NDT80* under the conditions tested [16]. Similarly, the zinc cluster transcription factor Upc2, which regulates the expression of ergosterol biosynthesis genes, also binds to the *MDR1* promoter, but it is required neither for the benomyl-induced upregulation of the efflux pump nor for its constitutive upregulation by a hyperactive Mrr1 [22]. Nevertheless, under certain environmental conditions Upc2 can act either as an activator or a repressor of *MDR1* expression [27]. Ndt80 may therefore also modulate *MDR1* and *CDR2* expression levels under other conditions than those used in our present study.

Apart from the role of Ndt80 in modulating gene expression in response to environmental signals, which may vary depending on the conditions, a more important question with respect to drug resistance was whether Ndt80 is required for the constitutive overexpression of genes mediating azole resistance in strains that have acquired activating mutations in the transcription factors Mrr1, Tac1, or Upc2. To address this issue, we compared the promoter activities of relevant target genes in strains expressing activated forms of the transcription factors in the presence and absence of Ndt80. Our results demonstrate that Ndt80 is not required for the constitutive overexpression of *MDR1*, *CDR1*/*CDR2*, and *ERG11* and the resulting increase in fluconazole resistance caused by hyperactive Mrr1, Tac1, and Upc2, respectively. In fact, strains expressing hyperactive Mrr1 or Tac1 even displayed somewhat higher fluconazole resistance in the absence of Ndt80 than in its presence. Besides the efflux pumps, additional genes are upregulated in strains containing gain-of-function mutations in Mrr1 or Tac1, and these genes also contribute to the increased drug resistance of such strains [22], [28]. Apparently, the overexpression of Mrr1 and Tac1 target genes overcomes defects of the *ndt80*Δ mutants which are responsible for their fluconazole hypersusceptibility, and the absence of Ndt80 augments the capacity of constitutively active Mrr1 and Tac1 to mediate fluconazole resistance.

Our finding that Ndt80 is dispensable for the constitutive overexpression of *MDR1*, *CDR1*/*CDR2*, and *ERG11* in strains with gain-of-function mutations in the zinc cluster transcription factors Mrr1, Tac1, and Upc2, respectively, also has potential practical implications. Combating drug resistance is an important goal to maintain the efficiency of currently used antifungal drugs, which could be achieved by inhibiting efflux pumps or their transcriptional regulators [29], [30]. As Mdr1 and Cdr1/Cdr2 belong to different classes of transporters, their activities probably cannot be blocked by the same inhibitor. Similarly, the two types of efflux pumps and the ergosterol biosynthesis genes are specifically regulated by distinct zinc cluster transcription factors. A common mechanism that is used by Mrr1, Tac1, and Upc2 could provide a target for the simultaneous inhibition of several transcriptional mechanisms of drug resistance. As Ndt80 binds to the promoters of the major azole resistance genes and, at least under some conditions, contributes to the azole-induced expression of *CDR1* and ergosterol biosynthesis genes, it seemed to be an attractive candidate for such an approach. However, as we have shown in this study, hyperactive forms of Mrr1, Tac1, and Upc2, which are often the cause of azole resistance in clinical *C. albicans* isolates, do not require Ndt80 to upregulate their target genes and thereby mediate drug resistance. Therefore, blocking Ndt80 would be of little use in attempts to override the azole resistance of clinical strains.

## Materials and Methods

### Strains and growth conditions

The *C. albicans* strains used in this study are listed in Table S1. All strains were stored as frozen stocks with 15% glycerol at −80°C and subcultured on YPD agar plates (10 g yeast extract, 20 g peptone, 20 g glucose, 15 g agar per liter) at 30°C. Strains were routinely grown in YPD liquid medium at 30°C in a shaking incubator. For selection of nourseothricin-resistant transformants, 200 µg/ml nourseothricin (Werner Bioagents, Jena, Germany) was added to YPD agar plates. To obtain nourseothricin-sensitive derivatives in which the *SAT1* flipper cassette was excised by FLP-mediated recombination, transformants containing the *NDT80* deletion cassette were grown overnight in YCB-BSA-YE medium (23.4 g yeast carbon base, 4 g bovine serum albumin, 2 g yeast extract per liter, pH 4.0) without selective pressure to induce the *SAP2* promoter controlling *caFLP* expression. Alternatively, strains containing a *SAT1* flipper cassette in which the *caFLP* gene is expressed from the *MAL2* promoter (as in plasmids pTAC1M1, pMRR1R3, pTAC1R3, and pUPC2R1) were grown overnight in YPM medium (10 g yeast extract, 20 g peptone, 20 g maltose per liter) instead of YCB-BSA-YE to induce the *MAL2* promoter. One hundred to 200 cells were then spread on YPD plates containing 10 µg/ml nourseothricin and grown for 2 days at 30°C. Nourseothricin-sensitive clones were identified by their small colony size and confirmed by restreaking on YPD plates containing 200 µg/ml nourseothricin as described previously [31]. For the induction of the *MDR1* and *CDR2* promoters, overnight cultures of the *GFP* reporter and parental strains were diluted 10^−2^ in 3 ml fresh YPD medium in glass tubes and grown for 3 h at 30°C. After adding 50 µg/ml benomyl or 10 µg/ml fluphenazine, the cultures were incubated for an additional hour and the fluorescence of the cells was quantified by fluorescence-activated cell sorter (FACS) analysis. For the detection of GFP by Western immunoblotting, cells were treated as described above, except that the cultures were grown in 50 ml volumes in Erlenmeyer flasks to obtain sufficient amounts of cells.

### Plasmid constructions

For the deletion of *NDT80*, we first generated a modified *SAT1* flipper cassette in which the *MAL2* promoter controlling *caFLP* expression was replaced by a 4 kb fragment containing the promoter of the *SAP2-1* allele of strain SC5314 [32]. For this purpose, a SalI site in the *caSAT1* marker of pSFS2 [31] was first removed by inserting an XhoI-PstI fragment containing the *caSAT1* marker from pNIM6 [33] instead of the corresponding SalI-PstI fragment in pSFS2, resulting in pSFS4. The *SAP2-1* upstream region was then amplified from genomic DNA of strain SC5314 with the primers SAP2P27 and IPCR1 (all primers used in this study are listed in Table S2). The PCR product was digested at the introduced BamHI and SalI sites and used to replace the *MAL2* promoter in pSFS4, thereby generating pSFS5. In contrast to previous versions of the *SAT1* flipper cassette, in which *caFLP* expression is driven from the leaky *MAL2* promoter or a shorter *SAP2* promoter fragment, *caFLP* expression is tightly controlled and efficiently inducible after integration of the modified *SAT1* flipper cassette from pSFS5 into the *C. albicans* genome. The *NDT80* upstream and downstream regions were amplified with the primer pairs NDT80-4/NDT80-5 and NDT80-6/NDT80-7, respectively, and the SacI/SacII- and XhoI/ApaI-digested PCR products were cloned on both sides of the *SAT1* flipper cassette of pSFS5 to generate pNDT80M3. For reintroduction of *NDT80* into *ndt80*Δ mutants, the *NDT80* coding region and ca. 0.5 kb of upstream and downstream sequences were amplified with the primers NDT80-4 and NDT80-compl. The PCR product was digested with SacI and SacII and used to replace the *NDT80* upstream region in pNDT80M3, resulting in pNDT80K1.

Plasmid pERG11G2 contains a *Candida*-adapted *GFP* reporter gene [34] under the control of the *ERG11* promoter [11]. Similar plasmids in which *GFP* expression is driven by the *MDR1*, *CDR1*, and *CDR2* promoters were constructed by substituting the upstream and downstream regions of the corresponding genes for the *ERG11* flanking sequences in pERG11G2, resulting in plasmids pMDR1G3, pCDR1G1, and pCDR2G3, respectively. The *MDR1* upstream and downstream regions were amplified with the primer pairs MDR1p5/MDR1p7 and MDR1-3/MDR1-4, the *CDR1* upstream and downstream regions were amplified with the primer pairs CDR1F/CDR1R and CDR29/CDR30, and the *CDR2* upstream and downstream regions were amplified with the primer pairs CDR2-5/CDR2-6 and CDR2-3/CDR2-4.

Plasmids pUPC2R1 and pMRR1R3, which were used to replace the *UPC2* and *MRR1* wild-type alleles by the *UPC2* ^G648D^ and *MRR1* ^P683S^ alleles, respectively, with the help of the *SAT1* flipper cassette, were described previously [11], [22]. To obtain an analogous cassette for introduction of the *TAC1* ^G980E^ allele, the *TAC1* downstream region was first amplified with the primers TAC1-6 and TAC1-7, and the XhoI/ApaI-digested PCR product was substituted for the *UPC2* downstream fragment in pUPC2R1, yielding pTAC1R1. The C-terminal part of the *TAC1* gene was then amplified with the primers TAC1-11 and TAC1hyp-2, thereby introducing a G2939A exchange that results in the G980E gain-of-function mutation in Tac1 [6]. The PCR product was digested with SacI/BamHI and substituted for the *UPC2* ^G648D^ allele in the SacI/BglII-digested pTAC1R1 to obtain pTAC1R3. A *TAC1* deletion cassette was generated by amplifying the *TAC1* upstream region with the primers TAC1-14 and TAC1-15 and substituting the SacI/SacII-digested PCR product for the *UPC2* sequences in pTAC1R1 to obtain pTAC1M1.

### Strain constructions

*C. albicans* strains were transformed by electroporation [35] with the following gel-purified linear DNA fragments: the SacI-ApaI fragments from pNDT80M3 and pTAC1M1 were used to delete the *NDT80* and *TAC1* genes, respectively, in strain SC5314. The SacI-ApaI fragment from pNDT80K1 was used to reintroduce a functional *NDT80* copy into *ndt80*Δ mutants. The SacI-ApaI fragments from pMRR1R3, pTAC1R3, and pUPC2R1 were used to introduce the hyperactive *MRR1* ^P683S^, *TAC1* ^G980E^, and *UPC2* ^G648D^ alleles, respectively, instead of the corresponding wild-type alleles in strain SC5314 and in the *ndt80*Δ mutants. The ApaI-SacI fragment from pERG11G2, the ApaI-SacII fragment from pCDR1G1, the KpnI-SacII fragment from pCDR2G3, and the XhoI-SacII fragment from pMDR1G3 were used to integrate the P*_ERG11_-GFP*, P*_CDR1_-GFP*, P*_CDR2_-GFP*, and P*_MDR1_-GFP* reporter fusions into the corresponding genomic loci in different strains (see Table S1). The correct integration of each construct was confirmed by Southern hybridization with gene-specific probes. The introduction of the P683S, G980E, and G648D mutations into the first and second alleles of *MRR1*, *TAC1*, and *UPC2*, respectively, of the transformants was confirmed by reamplification with specific primers and direct sequencing of the PCR products.

### Isolation of genomic DNA and Southern hybridization

Genomic DNA from *C. albicans* strains was isolated as described previously [31]. The DNA was digested with appropriate restriction enzymes, separated on a 1% agarose gel and, after ethidium bromide staining, transferred by vacuum blotting onto a nylon membrane and fixed by UV crosslinking. Southern hybridization with enhanced chemiluminescence-labeled probes was performed with the Amersham ECL™ Direct Nucleic Acid Labelling and Detection System (GE Healthcare UK Limited, Little Chalfont Buckinghamshire, UK) according to the instructions of the manufacturer.

### FACS analysis

Fluorescence-activated cell sorter (FACS) analysis was performed with a FACSCalibur cytometry system equipped with an argon laser emitting at 488 nm (Becton Dickinson, Heidelberg, Germany). Fluorescence was measured on the FL1 fluorescence channel equipped with a 530-nm band-pass filter. Twenty thousand cells were analyzed per sample and were counted at a flow rate of 500 cells per second. Fluorescence data were collected by using logarithmic amplifiers. The mean fluorescence (arbitrary values) was determined with CellQuest Pro (Becton Dickinson) software.

### Western immunoblotting

Cells were collected by centrifugation, washed in 2.5 ml breaking buffer (100 mM Tris-HCl [pH 7.5], 200 mM NaCl, 20% glycerol, 5 mM EDTA), and broken by vortexing for 10 min at 4°C with 500 µl 0.5 mm glass beads in 500 µl breaking buffer (100 mM Tris-HCl [pH 7.5], 200 mM NaCl, 20% glycerol, 5 mM EDTA, 4% Complete, EDTA-free Protease Inhibitor Cocktail [Roche Diagnostics GmbH, Mannheim, Germany], 0.1% β-mercaptoethanol). Samples were centrifuged at 13,000 rpm for 10 min at 4°C, the supernatant was collected, and the protein concentration was quantified using the Bradford protein assay. Extracts were heated for 10 min at 65°C, and equal amounts of protein of each sample were separated on an SDS-12% polyacrylamide gel. Proteins were transferred onto a nitrocellulose membrane with a Mini-Protean Tetra System (Bio-Rad, Munich, Germany) and stained with Ponceau S to control for equal loading. GFP was detected using rabbit monoclonal GFP antibody ab32146 (Abcam, Cambridge, UK) and anti rabbit HRP G-21234 (Invitrogen GmbH, Darmstadt, Germany) as first and secondary antibodies, respectively. A chemiluminescence detection system (GE Healthcare) was used for signal detection.

### Fluconazole susceptibility assays

The fluconazole susceptibilities of the strains were determined using a previously described microdilution method [36], except that the assay was performed in SD medium (6.7 g yeast nitrogen base without amino acids [YNB; BIO 101, Vista, Calif.], 20 g glucose, 0.77 g of complete supplement medium [CSM, BIO101]), because HR medium, which we used in previous studies, was not commercially available any more. Both media produced largely identical results.

## Supporting Information

Table S1 ***C. albicans*** **strains used in this study.**
(DOC)

Table S2 **Primers used in this study.**
(DOC)
