# Supplementary material for: The Transcription Factor Ndt80 Does Not Contribute to Mrr1-, Tac1-, and Upc2-Mediated Fluconazole Resistance in Candida albicans
Source: PLoS One. 2011 Sep 27;6(9):e25623. doi: 10.1371/journal.pone.0025623 (PMC3181345; doi:10.1371/journal.pone.0025623)
Supplement: Table S1 — C. albicans strains used in this study. (DOC) [file pone.0025623.s001.doc]

## Table S1. C. albicans strains used in this study

| **Strain** | **Parent** | **Relevant characteristics or genotype ^1^** | **Reference** |
| --- | --- | --- | --- |
| SC5314 |  | Wild-type reference strain | [1] |
|  |  |  |  |
| *mrr1*Δ, *ndt80*Δ, and *tac1*Δ mutants | | | |
| SCMRR1M4A and ‑B | SC5314 | *mrr1*Δ::*FRT*/*mrr1*Δ::*FRT* | [2] |
| SCNDT80M1A | SC5314 | *ndt80‑1*Δ::*SAT1-FLIP*/*NDT80‑2* | this study |
| SCNDT80M1B | SC5314 | *NDT80‑1*/*ndt80‑2*Δ::*SAT1-FLIP* | this study |
| SCNDT80M2A | SCNDT80M1A | *ndt80‑1*Δ::*FRT*/*NDT80‑2* | this study |
| SCNDT80M2B | SCNDT80M1B | *NDT80‑1*/*ndt80‑2*Δ::*FRT* | this study |
| SCNDT80M3A | SCNDT80M2A | *ndt80‑1*Δ::*FRT*/*ndt80‑2*Δ::*SAT1-FLIP* | this study |
| SCNDT80M3B | SCNDT80M2B | *ndt80‑1*Δ::*SAT1-FLIP*/*ndt80‑2*Δ::*FRT* | this study |
| SCNDT80M4A | SCNDT80M3A | *ndt80‑1*Δ::*FRT*/*ndt80‑2*Δ::*FRT* | this study |
| SCNDT80M4B | SCNDT80M3B | *ndt80‑1*Δ::*FRT*/*ndt80‑2*Δ::*FRT* | this study |
| SCNDT80MK1A | SCNDT80M4A | *NDT80-SAT1-FLIP*/*ndt80‑2*Δ::*FRT* | this study |
| SCNDT80MK1B | SCNDT80M4B | *ndt80‑1*Δ::*FRT*/*NDT80-SAT1-FLIP* | this study |
| SCNDT80MK2A | SCNDT80MK1A | *NDT80-FRT*/*ndt80‑2*Δ::*FRT* | this study |
| SCNDT80MK2B | SCNDT80MK1B | *ndt80‑1*Δ::*FRT*/*NDT80-FRT* | this study |
| SCTAC1M1A | SC5314 | *tac1‑1*Δ::*SAT1-FLIP*/*TAC1‑2* | this study |
| SCTAC1M1B | SC5314 | *TAC1‑1*/*tac1‑2*Δ::*SAT1-FLIP* | this study |
| SCTAC1M2A | SCTAC1M1A | *tac1‑1*Δ::*FRT*/*TAC1‑2* | this study |
| SCTAC1M2B | SCTAC1M1B | *TAC1‑1*/*tac1‑2*Δ::*FRT* | this study |
| SCTAC1M3A | SCTAC1M2A | *tac1‑1*Δ::*FRT*/*tac1‑2*Δ::*SAT1-FLIP* | this study |
| SCTAC1M3B | SCTAC1M2B | *tac1‑1*Δ::*SAT1-FLIP*/*tac1‑2*Δ::*FRT* | this study |
| SCTAC1M4A | SCTAC1M3A | *tac1‑1*Δ::*FRT*/*tac1‑2*Δ::*FRT* | this study |
| SCTAC1M4B | SCTAC1M3B | *tac1‑1*Δ::*FRT*/*tac1‑2*Δ::*FRT* | this study |
|  |  |  |  |
| Strains expressing *GFP* under the control of the *MDR1*, *CDR1*, *CDR2*, or *ERG11* promoter in a wild-type background | | | |
| SCMG3A and -B | SC5314 | *MDR1*/*mdr1*::P*_MDR1_-GFP-caSAT1* | this study |
| SCCG1A and -B | SC5314 | *CDR1*/*cdr1*::P*_CDR1_-GFP-caSAT1* | this study |
| SCCG3A and -B | SC5314 | *CDR2*/*cdr2*::P*_CDR2_-GFP-caSAT1* | this study |
| SCEG2A and -B | SC5314 | *ERG11‑1*/*erg11‑2*::P*_ERG11_-GFP-caSAT1* | [3] |
|  |  |  |  |
| Strains expressing *GFP* under the control of the *MDR1*, *CDR1*, *CDR2*, or *ERG11* promoter in an *ndt80*Δ background | | | |
| SCΔ*ndt80*MG3A | SCNDT80M4A | *ndt80‑1*Δ::*FRT*/*ndt80‑2*Δ::*FRT*  *MDR1*/*mdr1*::P*_MDR1_-GFP-caSAT1* | this study |
| SCΔ*ndt80*MG3B | SCNDT80M4B | *ndt80‑1*Δ::*FRT*/*ndt80‑2*Δ::*FRT*  *MDR1*/*mdr1*::P*_MDR1_-GFP-caSAT1* | this study |
| SCΔ*ndt80*CG1A | SCNDT80M4A | *ndt80‑1*Δ::*FRT*/*ndt80‑2*Δ::*FRT*  *CDR1*/*cdr1*::P*_CDR1_-GFP-caSAT1* | this study |
| SCΔ*ndt80*CG1B | SCNDT80M4B | *ndt80‑1*Δ::*FRT*/*ndt80‑2*Δ::*FRT*  *CDR1*/*cdr1*::P*_CDR1_-GFP-caSAT1* | this study |
| SCΔ*ndt80*CG3A | SCNDT80M4A | *ndt80‑1*Δ::*FRT*/*ndt80‑2*Δ::*FRT*  *CDR2*/*cdr2*::P*_CDR2_-GFP-caSAT1* | this study |
| SCΔ*ndt80*CG3B | SCNDT80M4B | *ndt80‑1*Δ::*FRT*/*ndt80‑2*Δ::*FRT*  *CDR2*/*cdr2*::P*_CDR2_-GFP-caSAT1* | this study |
| SCΔ*ndt80*EG2A | SCNDT80M4A | *ndt80‑1*Δ::*FRT*/*ndt80‑2*Δ::*FRT*  *ERG11‑1*/*erg11‑2*::P*_ERG11_-GFP-caSAT1* | this study |
| SCΔ*ndt80*EG2B | SCNDT80M4B | *ndt80‑1*Δ::*FRT*/*ndt80‑2*Δ::*FRT*  *erg11‑1*::P*_ERG11_-GFP-caSAT1*/*ERG11‑2* | this study |
|  |  |  |  |
| Strains expressing *GFP* under the control of the *MDR1* or *CDR2* promoter in *mrr1*Δ or *tac1*Δ mutants | | | |
| SCΔ*mrr1*MG3A | SCMRR1M4A | *mrr1*Δ::*FRT*/*mrr1*Δ::*FRT*  *MDR1*/*mdr1*::P*_MDR1_-GFP-caSAT1* | this study |
| SCΔ*mrr1*MG3B | SCMRR1M4B | *mrr1*Δ::*FRT*/*mrr1*Δ::*FRT*  *MDR1*/*mdr1*::P*_MDR1_-GFP-caSAT1* | this study |
| SCΔ*tac1*CG3A | SCTAC1M4A | *tac1‑1*Δ::*FRT*/*tac1‑2*Δ::*FRT*  *CDR2*/*cdr2*::P*_CDR2_-GFP-caSAT1* | this study |
| SCΔ*tac1*CG3B | SCTAC1M4B | *tac1‑1*Δ::*FRT*/*tac1‑2*Δ::*FRT*  *CDR2*/*cdr2*::P*_CDR2_-GFP-caSAT1* | this study |
|  |  |  |  |
| Strains expressing hyperactive *MRR1*, *TAC1*, and *UPC2* alleles in a wild-type background | | | |
| SCMRR1R34A and -B | SC5314 | *MRR1*^P683S^-*FRT*/*MRR1*^P683S^*-FRT* | [4] |
| SCTAC1R31A | SC5314 | *TAC1‑1*/*TAC1*^G980E^*-SAT1-FLIP* | this study |
| SCTAC1R31B | SC5314 | *TAC1*^G980E^*-SAT1-FLIP*/*TAC1‑2* | this study |
| SCTAC1R32A | SCTAC1R31A | *TAC1‑1*/*TAC1*^G980E^*-FRT* | this study |
| SCTAC1R32B | SCTAC1R31B | *TAC1*^G980E^*-FRT*/*TAC1‑2* | this study |
| SCTAC1R33A | SCTAC1R32A | *TAC1*^G980E^*-SAT1-FLIP*/*TAC1*^G980E^*-FRT* | this study |
| SCTAC1R33B | SCTAC1R32B | *TAC1*^G980E^*-FRT*/*TAC1*^G980E^*-SAT1-FLIP* | this study |
| SCTAC1R34A | SCTAC1R33A | *TAC1*^G980E^*-FRT*/*TAC1*^G980E^*-FRT* | this study |
| SCTAC1R34B | SCTAC1R33B | *TAC1*^G980E^*-FRT*/*TAC1*^G980E^*-FRT* | this study |
| SCUPC2R14A and -B | SC5314 | *UPC2*^G648D^-*FRT*/*UPC2*^G648D^-*FRT* | [3] |
|  |  |  |  |
| Strains expressing hyperactive *MRR1*, *TAC1*, and *UPC2* alleles in *ndt80*Δ mutants and complemented strains | | | |
| SCΔ*ndt80*MRR1R31A | SCNDT80M4A | *ndt80‑1*Δ::*FRT*/*ndt80‑2*Δ::*FRT*  *MRR1*/*MRR1*^P683S^-*SAT1-FLIP* | this study |
| SCΔ*ndt80*MRR1R31B | SCNDT80M4B | *ndt80‑1*Δ::*FRT*/*ndt80‑2*Δ::*FRT*  *MRR1*/*MRR1*^P683S^-*SAT1-FLIP* | this study |
| SCΔ*ndt80*MRR1R32A | SCΔ*ndt80*MRR1R31A | *ndt80‑1*Δ::*FRT*/*ndt80‑2*Δ::*FRT*  *MRR1*/*MRR1*^P683S^-*FRT* | this study |
| SCΔ*ndt80*MRR1R32B | SCΔ*ndt80*MRR1R31B | *ndt80‑1*Δ::*FRT*/*ndt80‑2*Δ::*FRT*  *MRR1*/*MRR1*^P683S^-*FRT* | this study |
| SCΔ*ndt80*MRR1R33A | SCΔ*ndt80*MRR1R32A | *ndt80‑1*Δ::*FRT*/*ndt80‑2*Δ::*FRT*  *MRR1*^P683S^-*SAT1-FLIP*/*MRR1*^P683S^-*FRT* | this study |
| SCΔ*ndt80*MRR1R33B | SCΔ*ndt80*MRR1R32B | *ndt80‑1*Δ::*FRT*/*ndt80‑2*Δ::*FRT*  *MRR1*^P683S^-*SAT1-FLIP*/*MRR1*^P683S^-*FRT* | this study |
| SCΔ*ndt80*MRR1R34A | SCΔ*ndt80*MRR1R33A | *ndt80‑1*Δ::*FRT*/*ndt80‑2*Δ::*FRT*  *MRR1*^P683S^-*FRT*/*MRR1*^P683S^-*FRT* | this study |
| SCΔ*ndt80*MRR1R34B | SCΔ*ndt80*MRR1R33B | *ndt80‑1*Δ::*FRT*/*ndt80‑2*Δ::*FRT*  *MRR1*^P683S^-*FRT*/*MRR1*^P683S^-*FRT* | this study |
| SCΔ*ndt80*MRR1R34K1A | SCΔ*ndt80*MRR1R34A | *NDT80-SAT1-FLIP*/*ndt80‑2*Δ::*FRT*  *MRR1*^P683S^-*FRT*/*MRR1*^P683S^-*FRT* | this study |
| SCΔ*ndt80*MRR1R34K1B | SCΔ*ndt80*MRR1R34B | *ndt80‑1*Δ::*FRT*/*NDT80-SAT1-FLIP*  *MRR1*^P683S^-*FRT*/*MRR1*^P683S^-*FRT* | this study |
| SCΔ*ndt80*MRR1R34K2A | SCΔ*ndt80*MRR1R34K1A | *NDT80-FRT*/*ndt80‑2*Δ::*FRT*  *MRR1*^P683S^-*FRT*/*MRR1*^P683S^-*FRT* | this study |
| SCΔ*ndt80*MRR1R34K2B | SCΔ*ndt80*MRR1R34K1B | *ndt80‑1*Δ::*FRT*/*NDT80-FRT*  *MRR1*^P683S^-*FRT*/*MRR1*^P683S^-*FRT* | this study |
| SCΔ*ndt80*TAC1R31A | SCNDT80M4A | *ndt80‑1*Δ::*FRT*/*ndt80‑2*Δ::*FRT*  *TAC1*^G980E^-*SAT1-FLIP*/*TAC1‑2* | this study |
| SCΔ*ndt80*TAC1R31B | SCNDT80M4B | *ndt80‑1*Δ::*FRT*/*ndt80‑2*Δ::*FRT*  *TAC1‑1*/*TAC1*^G980E^-*SAT1-FLIP* | this study |
| SCΔ*ndt80*TAC1R32A | SCΔ*ndt80*TAC1R31A | *ndt80‑1*Δ::*FRT*/*ndt80‑2*Δ::*FRT*  *TAC1*^G980E^-*FRT*/*TAC1‑2* | this study |
| SCΔ*ndt80*TAC1R32B | SCΔ*ndt80*TAC1R31B | *ndt80‑1*Δ::*FRT*/*ndt80‑2*Δ::*FRT*  *TAC1‑1*/*TAC1*^G980E^-*FRT* | this study |
| SCΔ*ndt80*TAC1R33A | SCΔ*ndt80*TAC1R32A | *ndt80‑1*Δ::*FRT*/*ndt80‑2*Δ::*FRT*  *TAC1*^G980E^-*FRT*/*TAC1*^G980E^-*SAT1-FLIP* | this study |
| SCΔ*ndt80*TAC1R33B | SCΔ*ndt80*TAC1R32B | *ndt80‑1*Δ::*FRT*/*ndt80‑2*Δ::*FRT*  *TAC1*^G980E^-*SAT1-FLIP*/*TAC1*^G980E^-*FRT* | this study |
| SCΔ*ndt80*TAC1R34A | SCΔ*ndt80*TAC1R33A | *ndt80‑1*Δ::*FRT*/*ndt80‑2*Δ::*FRT*  *TAC1*^G980E^-*FRT*/*TAC1*^G980E^-*FRT* | this study |
| SCΔ*ndt80*TAC1R34B | SCΔ*ndt80*TAC1R33B | *ndt80‑1*Δ::*FRT*/*ndt80‑2*Δ::*FRT*  *TAC1*^G980E^-*FRT*/*TAC1*^G980E^-*FRT* | this study |
| SCΔ*ndt80*TAC1R34K1A | SCΔ*ndt80*TAC1R34A | *NDT80-SAT1-FLIP*/*ndt80‑2*Δ::*FRT*  *TAC1*^G980E^-*FRT*/*TAC1*^G980E^-*FRT* | this study |
| SCΔ*ndt80*TAC1R34K1B | SCΔ*ndt80*TAC1R34B | *ndt80‑1*Δ::*FRT*/*NDT80-SAT1-FLIP*  *TAC1*^G980E^-*FRT*/*TAC1*^G980E^-*FRT* | this study |
| SCΔ*ndt80*TAC1R34K2A | SCΔ*ndt80*TAC1R34K1A | *NDT80-FRT*/*ndt80‑2*Δ::*FRT*  *TAC1*^G980E^-*FRT*/*TAC1*^G980E^-*FRT* | this study |
| SCΔ*ndt80*TAC1R34K2B | SCΔ*ndt80*TAC1R34K1B | *ndt80‑1*Δ::*FRT*/*NDT80-FRT*  *TAC1*^G980E^-*FRT*/*TAC1*^G980E^-*FRT* | this study |
| SCΔ*ndt80*UPC2R11A | SCNDT80M4A | *ndt80‑1*Δ::*FRT*/*ndt80‑2*Δ::*FRT*  *UPC2‑1*/*UPC2*^G648D^-*SAT1-FLIP* | this study |
| SCΔ*ndt80*UPC2R11B | SCNDT80M4B | *ndt80‑1*Δ::*FRT*/*ndt80‑2*Δ::*FRT*  *UPC2‑1*/*UPC2*^G648D^-*SAT1-FLIP* | this study |
| SCΔ*ndt80*UPC2R12A | SCΔ*ndt80*UPC2R11A | *ndt80‑1*Δ::*FRT*/*ndt80‑2*Δ::*FRT*  *UPC2‑1*/*UPC2*^G648D^-*FRT* | this study |
| SCΔ*ndt80*UPC2R12B | SCΔ*ndt80*UPC2R11B | *ndt80‑1*Δ::*FRT*/*ndt80‑2*Δ::*FRT*  *UPC2‑1*/*UPC2*^G648D^-*FRT* | this study |
| SCΔ*ndt80*UPC2R13A | SCΔ*ndt80*UPC2R12A | *ndt80‑1*Δ::*FRT*/*ndt80‑2*Δ::*FRT*  *UPC2*^G648D^-*SAT1-FLIP*/*UPC2*^G648D^-*FRT* | this study |
| SCΔ*ndt80*UPC2R13B | SCΔ*ndt80*UPC2R12B | *ndt80‑1*Δ::*FRT*/*ndt80‑2*Δ::*FRT*  *UPC2*^G648D^-*SAT1-FLIP*/*UPC2*^G648D^-*FRT* | this study |
| SCΔ*ndt80*UPC2R14A | SCΔ*ndt80*UPC2R13A | *ndt80‑1*Δ::*FRT*/*ndt80‑2*Δ::*FRT*  *UPC2*^G648D^-*FRT*/*UPC2*^G648D^-*FRT* | this study |
| SCΔ*ndt80*UPC2R14B | SCΔ*ndt80*UPC2R13B | *ndt80‑1*Δ::*FRT*/*ndt80‑2*Δ::*FRT*  *UPC2*^G648D^-*FRT*/*UPC2*^G648D^-*FRT* | this study |
| SCΔ*ndt80*UPC2R14K1A | SCΔ*ndt80*UPC2R14A | *NDT80-SAT1-FLIP*/*ndt80‑2*Δ::*FRT*  *UPC2*^G648D^-*FRT*/*UPC2*^G648D^-*FRT* | this study |
| SCΔ*ndt80*UPC2R14K1B | SCΔ*ndt80*UPC2R14B | *ndt80‑1*Δ::*FRT*/*NDT80-SAT1-FLIP*  *UPC2*^G648D^-*FRT*/*UPC2*^G648D^-*FRT* | this study |
| SCΔ*ndt80*UPC2R14K2A | SCΔ*ndt80*UPC2R14K1A | *NDT80-FRT*/*ndt80‑2*Δ::*FRT*  *UPC2*^G648D^-*FRT*/*UPC2*^G648D^-*FRT* | this study |
| SCΔ*ndt80*UPC2R14K2B | SCΔ*ndt80*UPC2R14K1B | *ndt80‑1*Δ::*FRT*/*NDT80-FRT*  *UPC2*^G648D^-*FRT*/*UPC2*^G648D^-*FRT* | this study |
|  |  |  |  |
| Strains containing P*_MDR1_-GFP*, P*_CDR1_-GFP*, P*_CDR2_-GFP*, or P*_ERG11_-GFP* reporter fusions and expressing hyperactive *MRR1*, *TAC1*, or *UPC2* alleles in a wild-type background | | | |
| SCMRR1R34MG3A | SCMRR1R34A | *MRR1*^P683S^-*FRT*/*MRR1*^P683S^-*FRT*  *MDR1*/*mdr1*::P*_MDR1_-GFP-caSAT1* | this study |
| SCMRR1R34MG3B | SCMRR1R34B | *MRR1*^P683S^-*FRT*/*MRR1*^P683S^-*FRT*  *MDR1*/*mdr1*::P*_MDR1_-GFP-caSAT1* | this study |
| SCTAC1R34CG1A | SCTAC1R34A | *TAC1*^G980E^-*FRT*/*TAC1*^G980E^-*FRT*  *CDR1*/*cdr1*::P*_CDR1_-GFP-caSAT1* | this study |
| SCTAC1R34CG1B | SCTAC1R34B | *TAC1*^G980E^-*FRT*/*TAC1*^G980E^-*FRT*  *CDR1*/*cdr1*::P*_CDR1_-GFP-caSAT1* | this study |
| SCTAC1R34CG3A | SCTAC1R34A | *TAC1*^G980E^-*FRT*/*TAC1*^G980E^-*FRT*  *CDR2*/*cdr2*::P*_CDR2_-GFP-caSAT1* | this study |
| SCTAC1R34CG3B | SCTAC1R34B | *TAC1*^G980E^-*FRT*/*TAC1*^G980E^-*FRT*  *CDR2*/*cdr2*::P*_CDR2_-GFP-caSAT1* | this study |
| SCUPC2R14EG2A | SCUPC2R14A | *UPC2*^G648D^-*FRT*/*UPC2*^G648D^-*FRT*  *ERG11‑1*/*erg11‑2*::P*_ERG11_-GFP-caSAT1* | [3] |
| SCUPC2R14EG2B | SCUPC2R14B | *UPC2*^G648D^-*FRT*/*UPC2*^G648D^-*FRT*  *erg11‑1*::P*_ERG11_-GFP-caSAT1*/*ERG11‑2* | [3] |
|  |  |  |  |
| Strains containing P*_MDR1_-GFP*, P*_CDR1_-GFP*, P*_CDR2_-GFP*, or P*_ERG11_-GFP* reporter fusions and expressing hyperactive *MRR1*, *TAC1*, or *UPC2* alleles in an *ndt80*Δ background | | | |
| SCΔ*ndt80*MRR1R34MG3A | SCΔ*ndt80*MRR1R34A | *ndt80‑1*Δ::*FRT*/*ndt80‑2*Δ::*FRT*  *MRR1*^P683S^-*FRT*/*MRR1*^P683S^-*FRT*  *MDR1*/*mdr1*::P*_MDR1_-GFP-caSAT1* | this study |
| SCΔ*ndt80*MRR1R34MG3B | SCΔ*ndt80*MRR1R34B | *ndt80‑1*Δ::*FRT*/*ndt80‑2*Δ::*FRT*  *MRR1*^P683S^-*FRT*/*MRR1*^P683S^-*FRT*  *MDR1*/*mdr1*::P*_MDR1_-GFP-caSAT1* | this study |
| SCΔ*ndt80*TAC1R34CG1A | SCΔ*ndt80*TAC1R34A | *ndt80‑1*Δ::*FRT*/*ndt80‑2*Δ::*FRT*  *TAC1*^G980E^-*FRT*/*TAC1*^G980E^-*FRT*  *CDR1*/*cdr1*::P*_CDR1_-GFP-caSAT1* | this study |
| SCΔ*ndt80*TAC1R34CG1B | SCΔ*ndt80*TAC1R34B | *ndt80‑1*Δ::*FRT*/*ndt80‑2*Δ::*FRT*  *TAC1*^G980E^-*FRT*/*TAC1*^G980E^-*FRT*  *CDR1*/*cdr1*::P*_CDR1_-GFP-caSAT1* | this study |
| SCΔ*ndt80*TAC1R34CG3A | SCΔ*ndt80*TAC1R34A | *ndt80‑1*Δ::*FRT*/*ndt80‑2*Δ::*FRT*  *TAC1*^G980E^-*FRT*/*TAC1*^G980E^-*FRT*  *CDR2*/*cdr2*::P*_CDR2_-GFP-caSAT1* | this study |
| SCΔ*ndt80*TAC1R34CG3B | SCΔ*ndt80*TAC1R34B | *ndt80‑1*Δ::*FRT*/*ndt80‑2*Δ::*FRT*  *TAC1*^G980E^-*FRT*/*TAC1*^G980E^-*FRT*  *CDR2*/*cdr2*::P*_CDR2_-GFP-caSAT1* | this study |
| SCΔ*ndt80*UPC2R14EG2A | SCΔ*ndt80*UPC2R14A | *ndt80‑1*Δ::*FRT*/*ndt80‑2*Δ::*FRT*  *UPC2*^G648D^-*FRT*/*UPC2*^G648D^-*FRT*  *ERG11‑1*/*erg11‑2*::P*_ERG11_-GFP-caSAT1* | this study |
| SCΔ*ndt80*UPC2R14EG2B | SCΔ*ndt80*UPC2R14B | *ndt80‑1*Δ::*FRT*/*ndt80‑2*Δ::*FRT*  *UPC2*^G648D^-*FRT*/*UPC2*^G648D^-*FRT*  *erg11‑1*::P*_ERG11_-GFP-caSAT1*/*ERG11‑2* | this study |

^1^ *SAT1-FLIP* denotes the *SAT1* flipper cassette; *FRT* is the FLP recombination target sequence, one copy of which remains in the genome after recycling of the *SAT1* flipper cassette; *caSAT1* is the *Candida*-adapted *SAT1* gene. The two alleles of *NDT80*, *TAC1*, *UPC2*, and *ERG11* in strain SC5314 were distinguished by EcoRV, SpeI, EcoRI, and HindIII restriction site polymorphisms, respectively. The *NDT80* allele containing the polymorphic downstream EcoRV site was arbitrarily designated *NDT80‑1*, the *TAC1* allele with the polymorphic downstream SpeI site *TAC1‑2*, the *UPC2* allele with the polymorphic EcoRI site at position +1593 *UPC2‑2*, and the *ERG11* allele with the polymorphic HindIII site at position +347 *ERG11‑2*.

**References**

1. Gillum AM, Tsay EY, Kirsch DR (1984) Isolation of the *Candida albicans* gene for orotidine-5'-phosphate decarboxylase by complementation of *S. cerevisiae ura3* and *E. coli pyrF* mutations. Mol Gen Genet 198: 179-182.

2. Morschhäuser J, Barker KS, Liu TT, Blaß-Warmuth J, Homayouni R, et al. (2007) The transcription factor Mrr1p controls expression of the *MDR1* efflux pump and mediates multidrug resistance in *Candida albicans*. PLoS Pathog 3: e164.

3. Heilmann CJ, Schneider S, Barker KS, Rogers PD, Morschhäuser J (2010) An A643T mutation in the transcription factor Upc2p causes constitutive *ERG11* upregulation and increased fluconazole resistance in *Candida albicans*. Antimicrob Agents Chemother 54: 353-359.

4. Schubert S, Barker KS, Znaidi S, Schneider S, Dierolf F, et al. (2011) Regulation of efflux pump expression and drug resistance by the transcription factors Mrr1, Upc2, and Cap1 in *Candida albicans*. Antimicrob Agents Chemother 55: 2212-2223.
