# Supplementary material for: The Transcription Factor Ndt80 Does Not Contribute to Mrr1-, Tac1-, and Upc2-Mediated Fluconazole Resistance in Candida albicans
Source: PLoS One. 2011 Sep 27;6(9):e25623. doi: 10.1371/journal.pone.0025623 (PMC3181345; doi:10.1371/journal.pone.0025623)
Supplement: Table S2 — Primers used in this study. (DOC) [file pone.0025623.s002.doc]

## Table S2. Primers used in this study

| **Primer** | **Sequence ^1^** |
| --- | --- |
| CDR1F | 5’-GATCGGGCCCTCGTTACTCAATAAGTAT-3’ |
| CDR1R | 5’-AGCTCTCGAGTTCTTTTTGACCTTTTAAAG-3’ |
| CDR29 | 5’-AATTCTGCAGTTTGTTTTTTGACATGGTGGTATC-3’ |
| CDR30 | 5’-TCGTGCCGCGGTTAGTATACGATAACTGTG-3’-3’ |
| CDR2-3 | 5’-AAAACTGCAGGATGGGGTCTTATTTTAC-3’ |
| CDR2-4 | 5’-CTGGGACCCTGCCGCGGTAATGATGTTG-3’ |
| CDR2-5 | 5’-AGATGGTACCAGGTAGTGATAGTTAACACACC-3’ |
| CDR2-6 | 5’-ATATGTCGACATTGTATGTGTTAATTAGTGAAATA-3’ |
| IPCR1 | 5’-TGTGTGTCGACAATGGTGATGTCTAGTGG-3’ |
| MDR1-3 | 5’-TGCTCTGCAGGAGGTGTCATTGTTTTCC-3’ |
| MDR1-4 | 5’-ATCACCGCGGGTTACAAGTATGACTGG-3’ |
| MDR1p5 | 5’-GCATTGTCGACGTTCTATGTAAGTAGATGTATTGC-3’ |
| MDR1p7 | 5’-CGTAAATCTCGAGAAACGGACTCCG-3’ |
| NDT80-4 | 5’-CATTAGAGCTCCTTTCCCATCTCCATATTACCA-3’ |
| NDT80-5 | 5’-AAACCCGCGGAAATGAGGAAGCTGGGACTGTT-3’ |
| NDT80-6 | 5’-GTTAATCTCGAGTGATGATGACGAAGAAAGGTTG-3’ |
| NDT80-7 | 5’-CATTAGGGCCCATGGGGGTGGATTGATCATT-3’ |
| NDT80-compl | 5’-AAACCCGCGGATGGGGGTGGATTGATCATT-3’ |
| SAP2P27 | 5’-ATATGGATCCTTGTCTCATTCTCTCGGTC-3’ |
| TAC1-6 | 5’-TAAACTCGAGTTGTAATTGGTGAAAGCG-3’ |
| TAC1-7 | 5’-TTTTGGGCCCTGGTGAAATTCCGAACC-3’ |
| TAC1-11 | 5’-AATTGAGCTCAGTTCAAGCAAGTACTGGC-3’ |
| TAC1-14 | 5’-TTAAGAGCTCGCAGTACATATAATAAAGTGGG-3’ |
| TAC1-15 | 5’-GGGTGAGTTCCCGCGGACAGTGAAGTGTCC-3’ |
| TAC1hyp-2 | 5’-TTTTGGATCCTTAAATCTCCAAATTATTGTCAAAG-3’ |

^1^ Introduced restriction sites are underlined.
